# Diversity in Biosynthetic Pathways of Galactolipids in the Light of Endosymbiotic Origin of Chloroplasts

**DOI:** 10.3389/fpls.2016.00117

**Published:** 2016-02-05

**Authors:** Naoki Sato, Koichiro Awai

**Affiliations:** ^1^Department of Life Sciences, Graduate School of Arts and Sciences, University of TokyoTokyo, Japan; ^2^Japan Science and Technology Agency, CRESTTokyo, Japan; ^3^Department of Biological Science, Faculty of Science, and Research Institute of Electronics, Shizuoka UniversityShizuoka, Japan

**Keywords:** cyanobacteria, galactolipids, glucolipid, photosynthesis, endosymbiosis

## Abstract

Cyanobacteria and chloroplasts perform oxygenic photosynthesis, and share a common origin. Galactolipids are present in the photosynthetic membranes of both cyanobacteria and chloroplasts, but the biosynthetic pathways of the galactolipids are significantly different in the two systems. In this minireview, we explain the history of the discovery of the cyanobacterial pathway, and present a probable scenario of the evolution of the two pathways.

## Introduction

Cyanobacteria perform oxygenic photosynthesis like chloroplasts of land plants and algae. The initial reactions of photosynthesis such as photochemical reactions, electron transport reactions, and ATP synthesis are performed in the thylakoid membranes, namely, flattened sac-like membranes specialized for photosynthesis. Thylakoid membranes are built up with galactolipids and acidic lipids. There are two major classes of galactolipids, monogalactosyl diacylglycerol (MGDG) and digalactosyl diacylglycerol (DGDG), which are both major components of all thylakoid membranes, in other words, typical of photosynthetic organisms. MGDG was shown to be required for normal development of chloroplasts (Kobayashi et al., 2007, 2013). The universality of galactolipids in photosynthetic membranes has been understood in terms of endosymbiotic theory, namely, that chloroplasts originated from cyanobacteria (see for example, Sato, 2001, 2006; Petroutsos et al., 2014).

**FIGURE 1:**
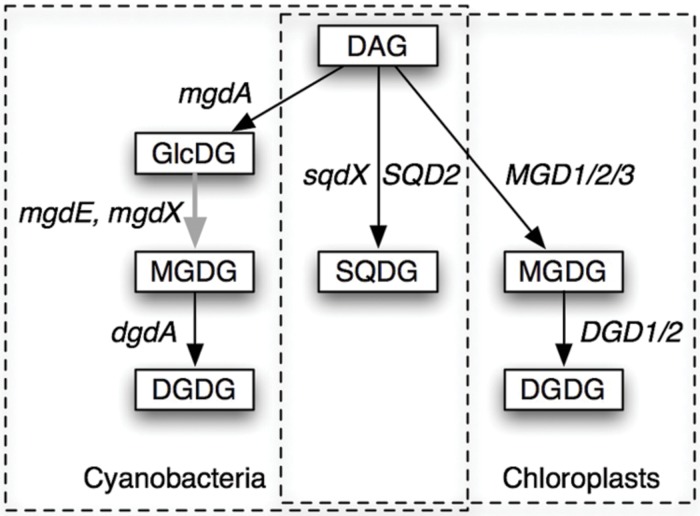
**Pathways of synthesis of glycolipids in cyanobacteria and chloroplasts.** The pathways and the genes involved in the processes are shown. DAG, diacylglycerol; GlcDG, monoglucosyl diacylglycerol; MGDG, monogalactosyl diacylglycerol; DGDG, digalactosyl diacylglycerol; SQDG, sulfoquinovosyl diacylglycerol. Gene names are explained in the text. *mgdX* is a hypothetical alternative gene encoding the epimerase. Solid arrows indicate glycosyltransferases (GT), while the gray arrow indicates an epimerase.

Biosynthesis of these galactolipids is, however, quite different in cyanobacteria and chloroplasts (**Figure 1**). In the chloroplasts of land plants, MGDG is synthesized by galactosylation of diacylglycerol (DAG), and DGDG is synthesized by the second galactosylation of MGDG (Shimojima and Ohta, 2011; Dörmann, 2013). In contrast, cyanobacteria have monoglucosyl diacylglycerol (GlcDG; Feige et al., 1980), which serves as a precursor to MGDG (Sato and Murata, 1982a). The conversion of GlcDG to MGDG was presumed to be epimerization, namely, the isomerization at the C-4 of the glucose moiety (Sato and Murata, 1982a). The glucosyltransferase activity was subsequently demonstrated (Sato and Murata, 1982b), while the enzymatic activity of the epimerase has never been detected *in vitro*. This was the reason why the epimerization hypothesis remained elusive until the identification of the responsible gene (Awai et al., 2014). Plant galactosyltransferases for the synthesis of MGDG and DGDG have been identified in the late 1990s and named MGD1 (Shimojima et al., 1997) and DGD1 (Dörmann et al., 1999), respectively, and homologs MGD2/3 and DGD2 were found later. In contrast, the enzymes involved in the synthesis of galactolipids in cyanobacteria have been uncharacterized until quite recently.

## Identification of the Enzymes/Genes for the Synthesis of Galactolipids

### Identification of Glycosyltransferases (GT)

Awai et al. (2006) identified the gene *mgdA* encoding the glucosyltransferase for the synthesis of GlcDG in *Synechocystis* sp. PCC 6803. The enzyme MgdA belongs to the glycosyltransferase (GT) family 2, and its domain structure was quite different from the plant galactosyltransferases MGD1/2/3, which belong to the GT 28, according to the CAZy database (Lombard et al., 2014). They also contain different InterPro domains (Mitchell et al., 2015). Homologs of MGD1 were found in green non-sulfur (GNS) bacteria (Yuzawa et al., 2012; **Supplementary Figure S1**; see Cluster 2103 of dataset Gclust2012_42 in comparative genomic database Gclust: Sato, 2009, available at http://gclust.c.u-tokyo.ac.jp/), but they are different from MgdA. Distant homologs of MgdA are found in α-proteobacteria (**Supplementary Figure S2**; see Gclust Cluster 2866).

**FIGURE 2:**
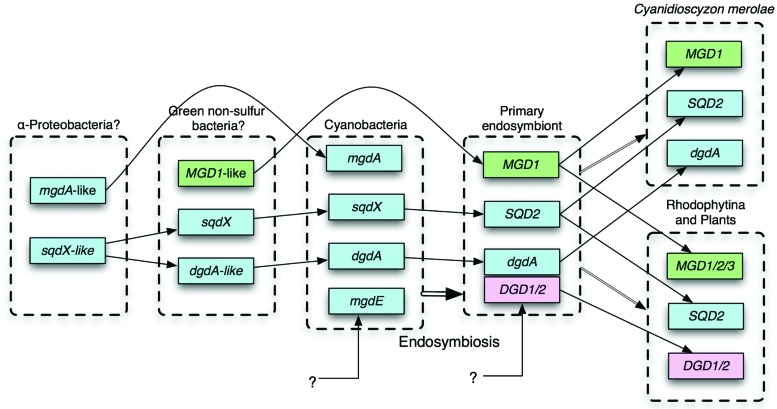
**Probable evolutionary scenario of glycolipid biosynthesis enzymes.** For details, see text. Cyan, genes identified in cyanobacteria; green, originated from green non-sulfur (GNS) bacteria; pink, unknown eukaryotic origin.

No homolog of *DGD1* had been detected in the sequenced cyanobacterial genomes or in the genome of *Cyanidioschyzon merolae*, which was the only red alga that was sequenced in the early 2000s. Cyanobacterial galactosyltransferase that catalyzes the synthesis of DGDG was identified by exploiting comparative genomics (Sato et al., 2005; Sato, 2009), which identified a GT that was conserved in cyanobacteria and *C. merolae* (Awai et al., 2007; Sakurai et al., 2007). The enzyme named DgdA has two InterPro domains, *N*-terminal Glycosyltransf_like_4 and *C*-terminal Glycosyltransf_1, whereas DGD1/2 has a single Glycosyltransf_1 domain. Both DgdA and DGD1//2 belong to the CAZy GT4 family. DGD1/2 has homologs in only plants and algae (Gluster 2454 in Gclust). It is interesting to note that DgdA is a close relative of SqdX/SQD2, an enzyme catalyzing the transfer of sulfoquinovose (**Figure 1**). This enzyme has a dual GT domain similar to that in DgdA. Phylogenetic analysis suggests that plant SQD2 originates from cyanobacterial SqdX, which is also found in various bacteria such as α-proteobacteria and GNS bacteria (**Supplementary Figure S3**). This points to a possibility that the gene *dgdA* originated from an *sqdX*-like gene before the emergence of cyanobacteria. Based on these considerations, it is likely that *mgdA* and *dgdA* originated from α-proteobacteria and GNS bacteria, respectively (**Figure 2**). Curiously, the SQDG synthesis pathway has been lost in *Gloeobacter violaceus*, which is the most deeply branching species in cyanobacteria.

### Identification of GlcDG Epimerase

The epimerase remained unidentified for a long time despite considerable efforts of many researchers. The use of Gclust, however, gave the clue again. Important additional information was given by the chromatophore genome of *Paulinella chromatophora*. The chromatophore of this microorganism looks like a chloroplast, but the sequencing of the chromatophore genome suggested that it originated from *Prochlorococcus*-like cyanobacteria (Nowack et al., 2008), but does not belong to the lineage of known chloroplasts, which are all monophyletic and originated from the deep root of cyanobacteria (Shih et al., 2013). The chromatophore genome encodes *mgdA*, *dgdA*, and *sqdX*. It was quite possible that an unknown GlcDG epimerase is also encoded in the genome. Comparative genomic analysis using Gclust indeed revealed that a putative membrane-bound oxidoreductase is conserved in most cyanobacteria and the chromatophore (Awai et al., 2014). Expression in *Escherichia coli* of the corresponding gene *sll1376* in *Synechocystis* sp. PCC 6803 demonstrated that it encodes an enzyme converting GlcDG to MGDG. Disruption of the gene in *Synechocystis* resulted in the cells in which all MGDG and DGDG were replaced by GlcDG. The gene was named *mgdE*. The enzyme MgdE consisted of an *N*-terminal hydrophobic domain assigned as the ‘fatty acid hydroxylase domain’ and a *C*-terminal oxidoreductase domain known as the ‘Rossmann-fold’ (Rao and Rossmann, 1973), thus considered as having a reasonable structure as a membrane-bound epimerase.

### Role of MgdE in Epimerization

The enzyme MgdE was conserved in many cyanobacteria, but curiously, not in all cyanobacteria. The gene *mgdE* was not detected in at least *G. violaceus*, *Thermosynechococcus elongatus*, and *Acariochloris marina*. In addition, the enzyme in various strains of *Prochlorococcus marinus*, marine *Synechococcus* species as well as *P. chromatophora* lacked the *N*-terminal domain. This raised again a fundamental question regarding the pathway of galactolipid synthesis in cyanobacteria, namely, if all cyanobacteria contain GlcDG as a precursor to MGDG. The detection of GlcDG has been especially difficult because it is a very minor component in many cyanobacteria. Even in a recently published review [**Figure 1** and Supplementary Table S1 in Petroutsos et al. (2014)], GlcDG was described as undetected in many cyanobacteria such as *Synechocystis* sp. PCC 6803 and *G. violaceus*.

This situation made it important to re-analyze GlcDG in the cyanobacteria in which *mgdE* was not detected or truncated. Sato (2015b) isolated and identified GlcDG in *G. violaceus*, *T. elongatus*, *A. marius* as well as in *P. marinus*. In addition, the conversion of GlcDG to MGDG was demonstrated by radiolabeling experiments in *G. violaceus* and *P. marinus*. Comparison of the composition of molecular species of GlcDG and MGDG also suggested that GlcDG can be considered as a precursor to MGDG in all the cyanobacteria analyzed, on the assumption that the molecular species containing saturated fatty acids are synthesized first and then the acyl groups are desaturated on the intact glycolipids while keeping the overall structure of lipids (Sato et al., 1986). This raised a question as to the universality of the role of *mgdE* in the epimerization of GlcDG in cyanobacteria. Is there another GlcDG epimerase in some cyanobacteria? In that case, how was the pathway of galactolipid synthesis acquired in the cyanobacteria?

MgdE belongs to a large family of bifunctional sterol desaturases/short-chain dehydrogenases (Kramm et al., 2012), including various enzymes related to lipid metabolism such as FabG, 3-oxoacyl-ACP reductase, involved in fatty acid biosynthesis [**Supplementary Figure S4** in Sato (2015b)]. In this respect, other members of this family could act as GlcDG epimerase (encoded by a hypothetical gene *mgdX* in **Figure 1**). This will be a new perspective of MGDG synthesis in cyanobacteria.

## Evolution of Galactolipid Biosynthesis

### Phylogenetic Distribution of the Cyanobacterial Pathway in Algae

As described above, the pathway of galactolipid biosynthesis is significantly different in cyanobacteria and chloroplasts of land plants. An immediate question arises as to what the situation is in algae. The chloroplasts of Archaeplastida (green plants, red algae, and glaucophytes) are monophyletic and are believed to originate from a single endosymbiotic event (Sato, 2001, 2006; Nowack et al., 2008; Shih et al., 2013). A survey of the Gclust database shows that the green algae, such as *Chlamydomonas reinhardtii* and *Ostreococcus tauri*, have a pathway consisting of MGD1 and DGD1, like land plants. The situation in red algae is complicated. *Cyanidioschyzon merolae* has a plastid-encoded *dgdA*, but has a copy of *MGD1*. Curiously, an *mgdA* homolog is also encoded in the *C. merolae* genome, although no GlcDG was detected by careful analysis (Sato and Moriyama, 2007). The *dgdA* gene (also known as *ycf82*) is also found in the plastid genomes of Cyanidiales algae, *Galdieria sulphuraria* and *Cyanidium caldarium*. This is not the case in another red alga *Porphyridium purpureum*, in which *dgdA* is not encoded in the plastid genome (Tajima et al., 2014), but a putative *DGD1* is encoded in the nuclear genome (Bhattacharya et al., 2013). The same is true for other red algae in Rhodophytina (Awai, 2015), which are unicellular or macrophytic red algae belonging to non-Cyanidiales clades. The glaucophyte *Cyanophora paradoxa* encodes *MGD1* and *dgdA* (Awai, 2015). The heterokonts such as diatoms are supposed to originate from a red algal secondary endosymbiosis, but none of them encodes *dgdA*.

## Concluding Remarks: Probable Evolutionary Scenarios

Comparative genomics clearly shows that all eukaryotes have MGD1 whereas all cyanobacteria have MgdA. Although there could be at least two different entities of GlcDG epimerase, all cyanobacteria are likely to synthesize MGDG by epimerization of GlcDG. In this respect, the replacement of MgdA–MgdE system by green bacterial MGD1 accompanied the primary endosymbiosis (**Figure 2**), which also accompanied drastic changes in the transcriptional and genomic machineries (Sato, 2001): Namely, prokaryotic transcription factors and DNA-binding proteins are not conserved in the chloroplasts, while the sigma factors become encoded by the nucleus.

In contrast, the replacement of DgdA by DGD1 seemed to occur at least two times, namely, during the evolution of green algae on the one hand, and during the evolution of Rhodophytina in the red algae on the other hand (Sato, 2015a), because all red algae are monophyletic (Tajima et al., 2014). Another, more plausible possibility implies that the *dgdA* gene and the *DGD1* gene coexisted in a hypothetical primary endosymbiont, and one of them was lost subsequently in different lineages (**Figure 2**). This is more likely because all DGD1 in both green plants and red algae are monophyletic (**Supplementary Figure S3**). A simple question should be asked: what is the functional difference between DgdA and DGD1, and why the two types of enzymes are present? To answer the question, we will have to know the enzymology and three-dimensional structure of the respective enzymes. In addition, it is clear that a more extensive survey of all algal phyla will be necessary to reveal the whole picture of the distribution of the two pathways, and hence the evolution of the DGDG biosynthesis.

## Author Contributions

Both authors contributed to all parts of collaborative works, including experiments and writing.

## Conflict of Interest Statement

The authors declare that the research was conducted in the absence of any commercial or financial relationships that could be construed as a potential conflict of interest.
