# Supplementary material for: Diversity in Biosynthetic Pathways of Galactolipids in the Light of Endosymbiotic Origin of Chloroplasts
Source: Front Plant Sci. 2016 Feb 5;7:117. doi: 10.3389/fpls.2016.00117 (PMC4742570; doi:10.3389/fpls.2016.00117)
Supplement: Supplementary file 1 [file Data_Sheet_1.PDF]

Supplementary Material

Diversity in biosynthetic pathways of galactolipids in the light of endosymbiotic origin of chloroplasts

Naoki Sato[1,2]*, Koichiro Awai[3,4]

*** Correspondence:** Naoki Sato: naokisat@bio.c.u-tokyo.ac.jp

1 Supplementary Figures

We provide three phylogenetic trees to make clear the description in the text. These are not considered as original data, but the secondary data derived from the sequence database, mostly Gclust database.

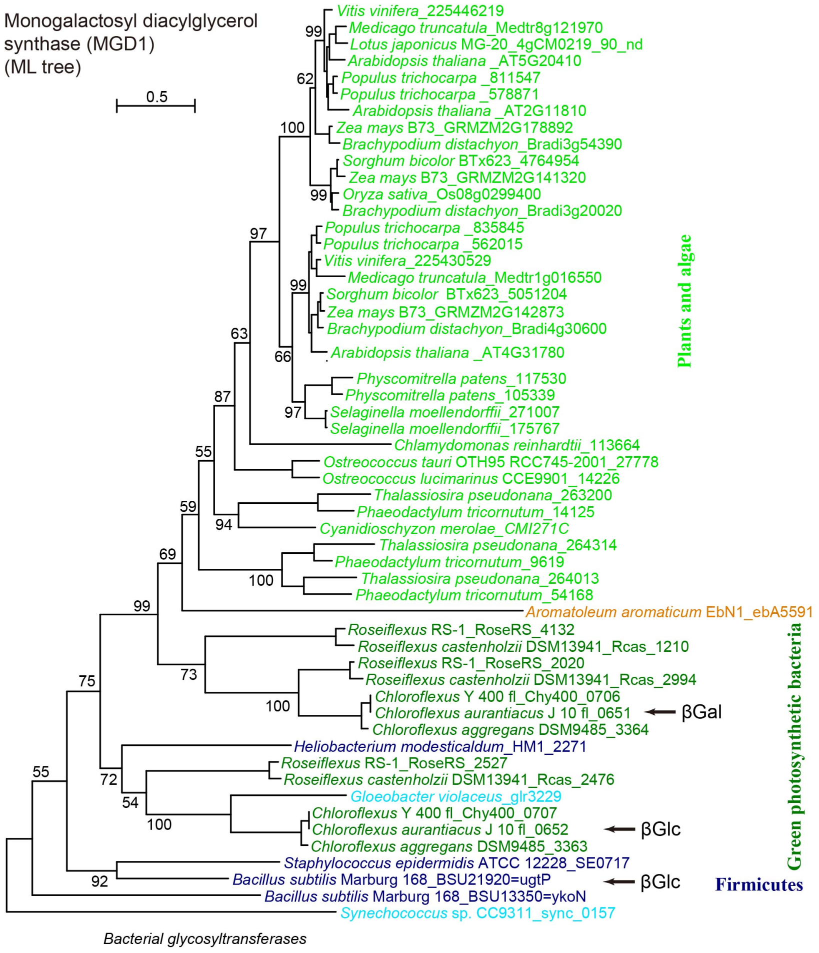

Supplementary Figure S1. Phylogenetic tree of MGD1. This is a maximum likelihood tree using the LG model. The sequences were obtained from the Gclust dataset 2012_42. The number on each branch indicates a confidence value. The bar indicates the measure of phylogenetic distance. Note that the enzymatic properties of some bacterial enzymes were identified as shown (Hölzl et al. 2005). Plant and algal MGD1/2/3 enzymes are β galactosyltransferases.

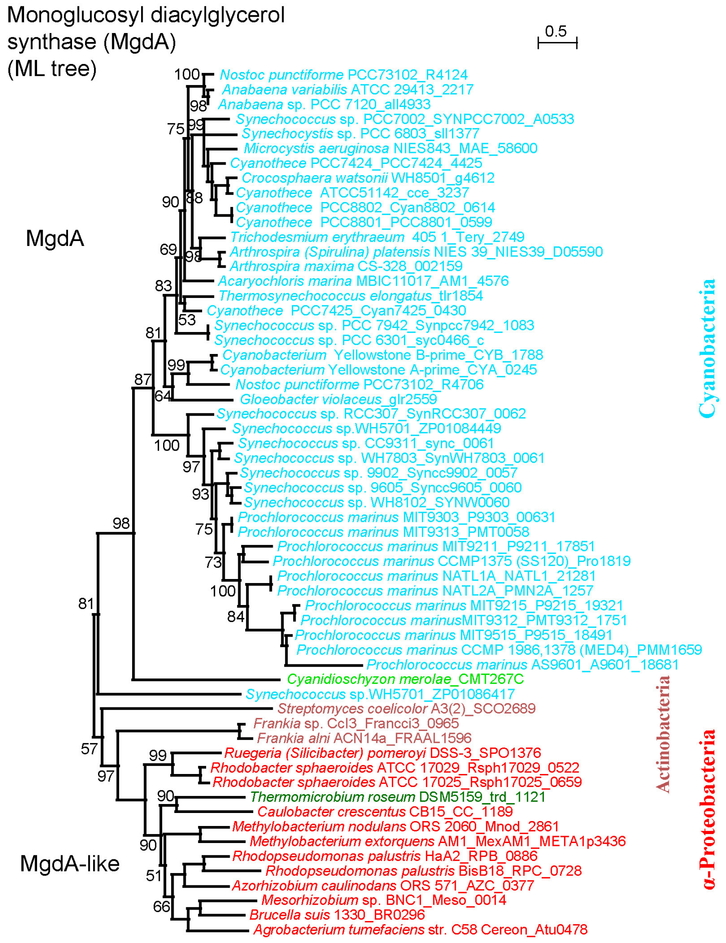

Supplementary Figure S2. Phylogenetic tree of MgdA. This is a maximum likelihood tree using the LG model. The sequences were obtained from the Gclust dataset 2012_42. The number on each branch indicates a confidence value. The bar indicates the measure of phylogenetic distance. Note that glycolipids with β-linkage were detected in *Rhodobacter sphaeroides* (Benning et al., 1995) and *Rhodopseudomonas viridis* (Linscheid et al., 1997), indicating that the α-proteobacterial MgdA-like homologs might be involved in synthesizing some of these glycolipids.

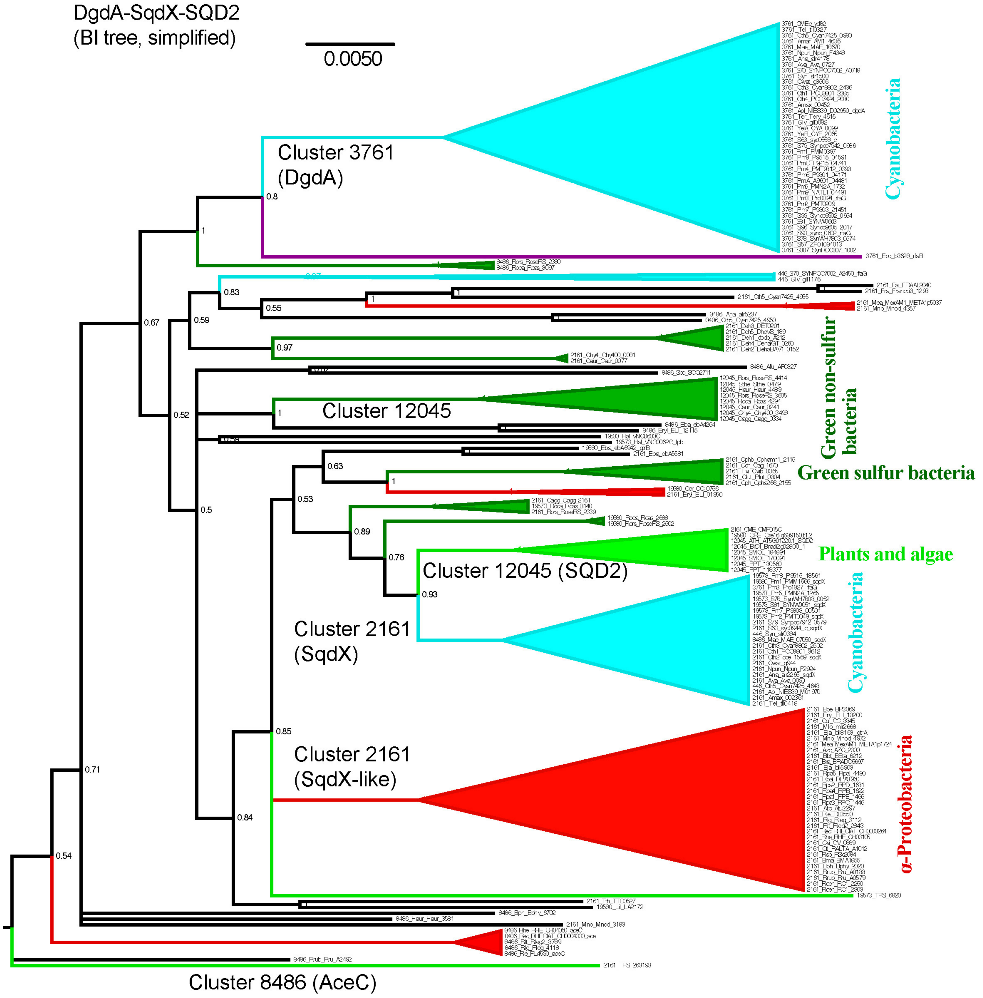

Supplementary Figure S3. Phylogenetic tree of DgdA and SqdX/SQD2. This is a Bayes inference tree using the WAG model. The sequences were obtained from the Gclust dataset 2012_42. Cluster numbers refer to the Gclust Clusters. The sequence names are intentionally shown in small characters to show the general shape of tree. Each sequence name consists of cluster number, organism ID, and gene ID, which are described in the Gclust database. The number on each branch indicates a confidence value. The bar indicates the measure of phylogenetic distance. Note that not all α-proteobacteria contain SQDG (Tsukatani et al., 2015). Green sulfur bacteria do not contain SQDG, but rhamnosyl diacylglycerol (RGDG) and MGDG. Green non-sulfur bacteria contain SQDG and GlcGalDG as well as MGDG. None of the green bacteria contain DGDG. Some of the members are likely to be involved in the synthesis of RGDG and GlcGalDG. Note also that the sulfoquinovosyltransferase SqdD of *Rhodobacter sphaeroides* belongs to GT8 (Gclust Cluster 1633), but not GT4 that is shown in this figure.

References

Benning, C., Huang, Z.-H. and Gage, D. A. (1995) Accumulation of a novel glycolipid and a betaine lipid in cells of Rhodobacter sphaeroides grown under phosphate limitation. *Arch. Biochem. Biophys*. 317, 103-111.

Hölzl, G., Zähringer, U., Warnecke, D. and Heinz, E. (2005) Glycoengineering of cyanobacterial thylakoid membranes for future studies on the role of glycolipids in photosynthesis. *Plant Cell Physiol.* 46, 1766-1778.

Leinscheid, M., Diehl, B. W. K., Övermöhle, M., Riedl, I. and Heinz, E. (1997) Membrane lipids of Rhodopseudomonas viridis. *Biochim. Biophys. Acta* 1347, 151-163.

Tsukatani, Y., Tamiaki, H. and Mizoguchi, T. (2015) Lipids in anoxygenic photosynthetic bacteria. *News Letter of the Japanese Society of Photosynthesis Research.* 25, 151-159 (in Japanese).
